# 590. Persisting COVID-19 vaccination hesitancy in the South Bronx

**DOI:** 10.1093/ofid/ofab466.788

**Published:** 2021-12-04

**Authors:** Danelly Gomez D' Aza, Masood A Shariff, Israel Duran Santibanez, Raquel Horowitz, Hina Asad, Dennis Mensah, Lara Rabiee, Anjana Pillai, Vipul Shah, Vidya Menon

**Affiliations:** 1 NYC HHC Lincoln, Bronx, New York; 2 Lincoln Medical Center, New York, New York

## Abstract

**Background:**

Minority groups have the lowest vaccination rates when compared to the overall population. We aim to study the attitudes and perceptions of COVID-19 vaccination, about six months after vaccine rollout in the South Bronx.

**Methods:**

Cross-sectional anonymized online survey evaluating knowledge, attitude and perception about COVID-19 vaccination using SurveyMonkey™ was conducted in South Bronx community from April - June 2021.

**Results:**

Of the 281 participants, 67% were Latinx and 16% were African American (AA); 69% (195) were fully vaccinated (FV) and 31% (86) with vaccine hesitancy (VH). The common reasons for hesitancy were “concerns about side effects” (38%), “vaccine is not safe” (27%) and “vaccine was approved too fast” (26%) (p< .001). VH were more likely to rely online/mobile apps (30%) and friends and family (23%) as compared to FV. VH were more likely to be AA, younger age (< 35 yrs), high school or lower education, single, unemployed, without comorbidities, not current on other eligible vaccines, and did not believe “vaccine is necessary to end the pandemic.” Majority of participants from both cohorts trusted their primary care providers. Mistrust with healthcare and pharmaceutical companies was higher in VH (p=0.009). Both groups preferred to continue wearing mask and practice social distancing despite vaccination status.

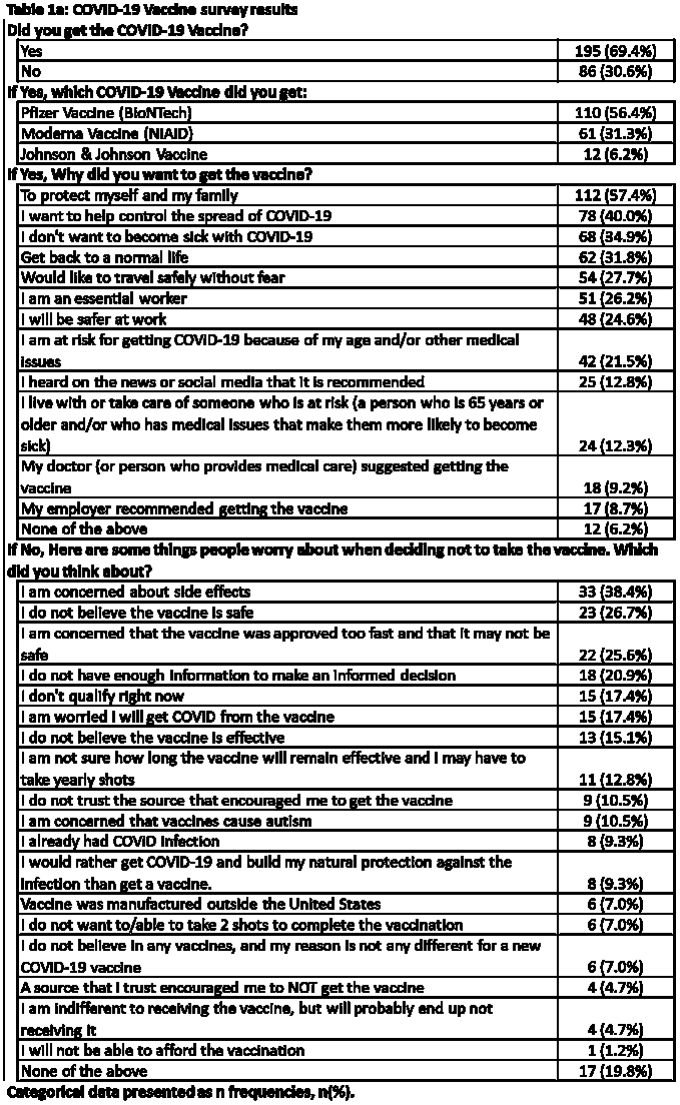

Table 1b: COVID-19 Vaccine Survey Summary

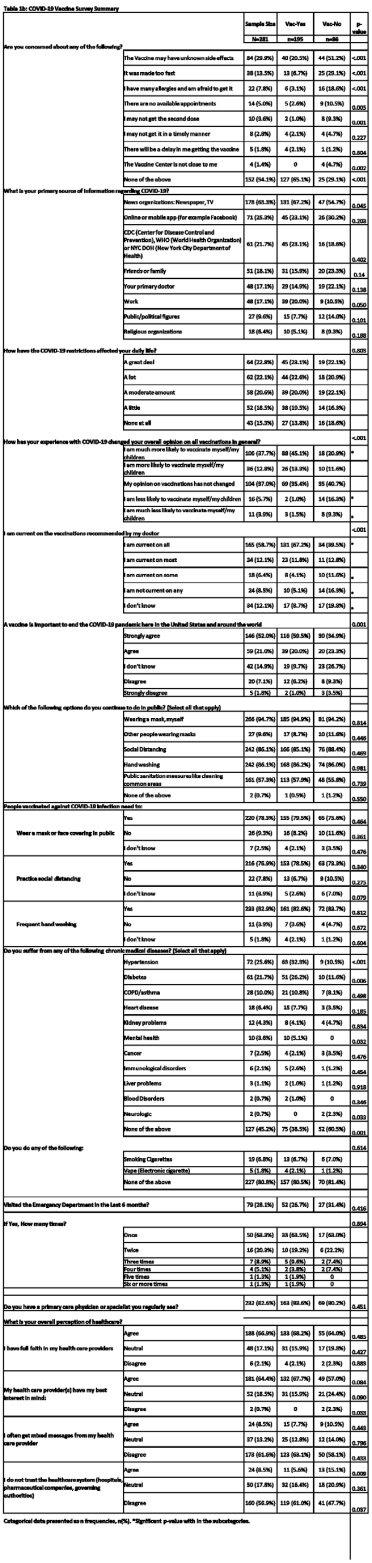

Table 1c: COVID-19 Vaccine Survey Participant Characteristics

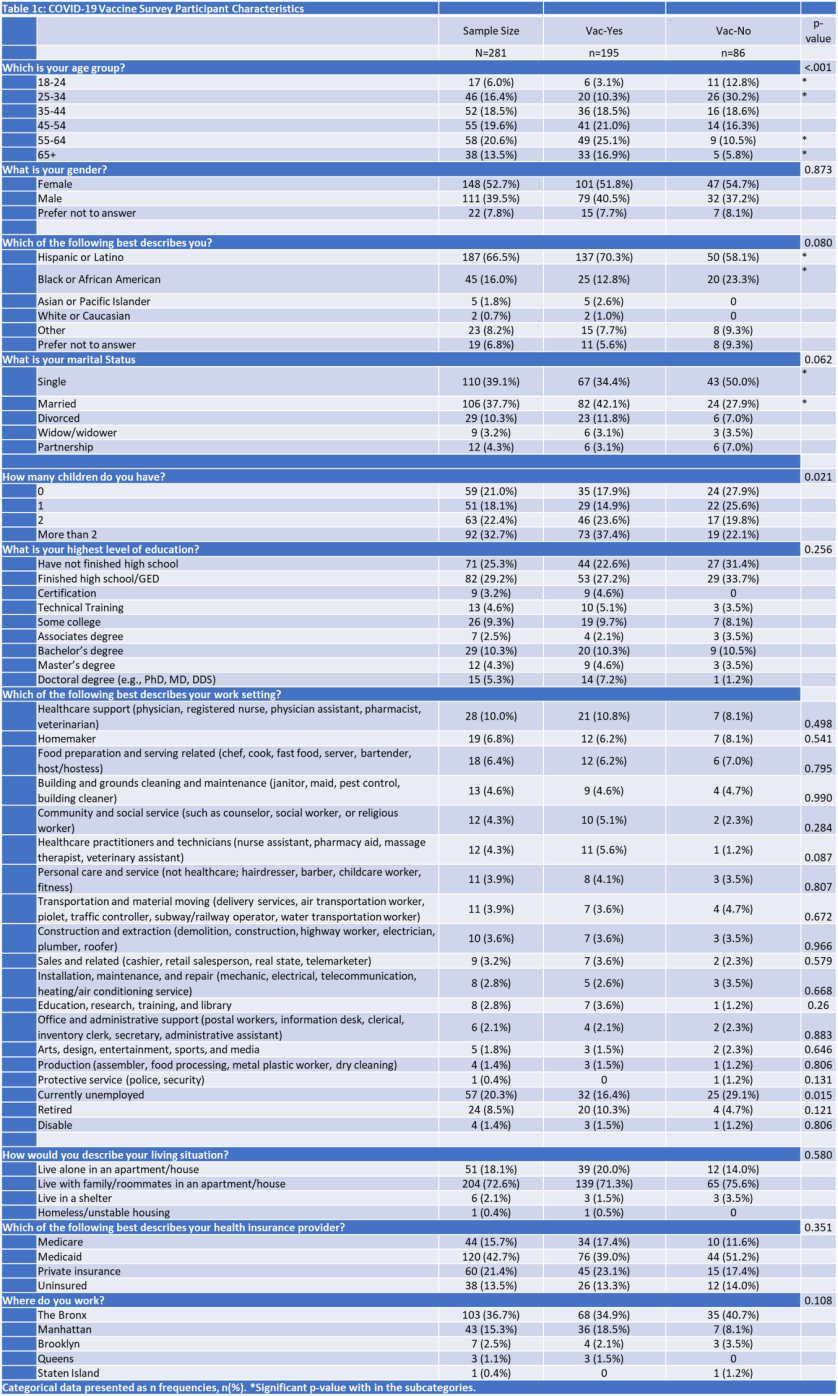

**Conclusion:**

Persisting vaccine hesitancy is concerning in minority communities. Identifying the target population and implementation of innovative methods to improve COVID-19 vaccination acceptance leveraging primary care providers would be a possible solution.

**Disclosures:**

**All Authors**: No reported disclosures

